# The Heat Is On: Effects of Synchronous Music on Psychophysiological Parameters and Running Performance in Hot and Humid Conditions

**DOI:** 10.3389/fpsyg.2018.01114

**Published:** 2018-07-10

**Authors:** Luke Nikol, Garry Kuan, Marilyn Ong, Yu-Kai Chang, Peter C. Terry

**Affiliations:** ^1^Exercise and Sports Science, School of Health Sciences, Universiti Sains Malaysia, Kota Bharu, Malaysia; ^2^Sports Science Unit, School of Medical Sciences, Universiti Sains Malaysia, Kota Bharu, Malaysia; ^3^Graduate Institute of Athletics and Coaching Science, National Taiwan Sport University, Taoyuan City, Taiwan; ^4^Division of Research and Innovation, University of Southern Queensland, Toowoomba, QLD, Australia

**Keywords:** synchronous music, psychophysiology, running, heat, humidity

## Abstract

Running in high heat and humidity increases psychophysiological strain, which typically impairs running performance. Listening to synchronous music has been shown to provide psychophysiological benefits, which may enhance running performance. The present randomized, crossover study examined effects of listening to synchronous music on psychophysiological parameters and running performance in hot and humid conditions. Twelve male runners (21.7 ± 2.2 y; 166.17 ± 7.18 cm; 60.32 ± 9.52 kg; 59.29 ± 5.95 ml kg^−1^ min^−1^) completed two running trials in simulated conditions (31°C and 70% humidity) with and without synchronous music. Participants ran on a treadmill inside a climatic chamber for 60 min at 60% $\dot{\text{V}}$O_2_max and continued to run to exhaustion at 80% $\dot{\text{V}}$O_2_max. Time-to-exhaustion under the synchronous music condition was 66.59% longer (mean = 376.5 s vs. 226.0 s, *p* = 0.02, *d* = 0.63) compared to the no music condition. Ratings of perceived exertion were significantly lower for the synchronous music condition at each time point (15, 30, 45, and 60 min) of the steady state portion of the running trials. Small differences in heart rate were detected between conditions. No significant between-condition differences were found in urine specific gravity, percentage of body weight loss, thermal comfort, and blood lactate. Findings suggest that listening to synchronous music is beneficial to running performance and perceived exertion in hot and humid conditions.

## Introduction

Listening to music while engaging in physical activity is a common practice for legions of athletes and exercise participants. A substantial body of empirical evidence has shown that music has the potential to produce a range of beneficial effects in the sport and exercise domain (Terry and Karageorghis, 2011). Benefits of music include positive emotional responses, such as feeling energized (e.g., Karageorghis and Jones, 2014; Hutchinson et al., 2018), reduced perceived exertion (e.g., Lim et al., 2014; Ruscello et al., 2014), improved performance (e.g., Terry et al., 2012; Bood et al., 2013), and greater physiological efficiency (e.g., Szmedra and Bacharach, 1998; Bacon et al., 2012).

Several studies have investigated the effects on endurance performance of synchronous music, where participants perform repetitive movements (e.g., walking, running, cycling) in time with the rhythmical elements of the music such as the beat or tempo. For example, Terry et al. (2012) tested synchronous music effects on treadmill running among elite triathletes. Time-to-exhaustion was 18.1 and 19.7% longer when running in time to motivational and neutral music, respectively, compared to no music. Motivational music typically has a fast tempo (> 120 beats per minute [bpm]), a strong rhythm, inspiring lyrics and an uplifting harmonic structure, which collectively tend to increase energy and induce bodily action. By contrast, neutral music does not have these characteristics but is not regarded as demotivational. Karageorghis et al. (2009) similarly compared motivational synchronous music, neutral synchronous music and no music during treadmill walking to exhaustion. Music condition accounted for 38% of the variance in endurance time, with motivational music associated with 15% longer endurance time over no music and 6% over neutral music. Bood et al. (2013) also showed that time-to-exhaustion during treadmill running was significantly longer with synchronous motivational music than without. Indeed, use of a simple metronome was associated with significantly better performance than no music, suggesting that any acoustic stimuli with a consistent beat that matches the rhythm of the activity may assist participants to synchronize their running stride to the tempo of the music, which seemingly increases effort and improves running economy.

In tropical countries such as Malaysia, athletes and exercisers are physically active for prolonged periods in hot, humid conditions, typically resulting in impaired physical performance (Tatterson et al., 2000; Saat et al., 2005). Hydration status is an important factor in detecting hypohydration and preventing performance deficits, such as increased perceived exertion, decreased time to fatigue, and increased thermal and cardiovascular strain (Minton et al., 2015). Saat et al. (2005) assessed the challenges to thermoregulation and blood parameters of running in tropical conditions, demonstrating significantly increased dehydration rate and decreased resting hemoglobin, hematocrit and red blood cells among runners completing a daily 60-min run over a 14-day period. Further, a study of the Australian National Road Cycling Squad, comparing performance in a 30-min time-trial, with (32°C) and without (23°C) heat stress, reported 6.5% reduced power output, higher skin temperature and higher sweat rate in the heat condition overall, with lower blood lactate and higher pH in the latter stages of the trial (Tatterson et al., 2000).

The effects of listening to synchronous music while exercising in the heat have not yet been established empirically. Therefore, in the present study, we assessed running performance in hot, humid conditions with synchronous music compared to no music, and examined whether listening to synchronous music affected blood lactate, heart rate (HR), thermal comfort, perceived exertion, and hydration status of recreational athletes.

## Materials and Methods

### Participants

Twelve healthy male participants (mean age = 21.7 ± 2.2 y) who ran at least 3 days per week were recruited as participants. Smokers and those with respiratory infections were excluded. Body mass index (BMI) of participants was 21.83 ± 2.96 kg m^−2^. Maximal oxygen uptake ($\dot{\text{V}}$O_2_max) of participants assessed at baseline was 59.29 ± 5.95 ml kg^−1^ min^−1^. Baseline $\dot{\text{V}}$O_2_max data were used to calculate running intensity for each participant during the experimental trials. The Physical Activity Readiness Questionnaire (PAR-Q; Gledhill, 2002), informed consent and participant demographic sheet were completed prior to participation. The required sample size was estimated using G-Power Version 3.1 (Faul et al., 2007). Based on a repeated-measures ANOVA with 2 running conditions (synchronous music and no music) x 4 time points (baseline, mid [30 and 60 min], post), statistical power set at 80% with a 95% confidence interval, and an effect size of 0.64 (Terry et al., 2012; $\dot{\text{V}}$O_2_max), a sample of 12 participants was judged to be sufficient to detect the hypothesized between-condition differences.

### Measures and Materials

#### Rating of Perceived Exertion and Thermal Comfort

The Rating of Perceived Exertion (RPE) scale (Borg, 1998) was used to assess the intensity of physical work as perceived by participants during the two trials, on a scale from 6 (no exertion) to 20 (maximal exertion). RPE was recorded pre-task, in-task (30 and 60 min), and immediately after the run to exhaustion. Thermal comfort was recorded pre-task and in-task (30 and 60 min) using the American Society of Heating, Refrigerating and Air-Conditioning Engineers (1966) (ASRHAE) Standard 55 (see also Epstein and Moran, 2006) which ranges from −3 (very cold) to +3 (very hot).

#### Music Selection

A shortlist of 20 music tracks with potential to be synchronized to individual running stride (1 or 2 strides per beat) was established using the protocol recommended by Karageorghis (2008) and rated for motivational qualities (rhythm, style, melody, tempo, sound and beat) by 10 health science undergraduate students using the Brunel Music Rating Inventory-3 (BMRI-3; Karageorghis, 2008). Music tracks with BMRI-3 ratings of 36–42, indicating motivational qualities, were shortlisted. Participants chose their preferred music selection from the shortlist, which were then assessed for synchronicity with running stride and small adjustments made to tempo (≤ 4 bpm) using the Virtual DJ software. **Table 1** includes the 20 music tracks used in this study. As an example, if a participant with a running cadence of 156 strides per minute chose Pump It by the Black Eyed Peas (bpm = 77) as preferred music, then the music tempo was raised to 78 bpm to allow the participant to run in synchrony with the music at two strides per beat.

**Table 1:** List of the 20 music tracks used.

Track Title	Artist	BPM
We Will Rock You	Five	91
Pump It	The Black Eyed Peas	77
Hurricane	Scorpions and Berlin Philharmonic	120
Gemuruh	Faizal Tahir	79
Gonna Fly Now (Theme from Rocky)	Bill Conti	81
Extravaganza	Bunkface	162
Hall Of Fame	William feat. The Script	85
Mentera Semerah Padi	M.Nasir and Spider	77
Gemuruh Suara	Team Malaysia’s Theme Song	148
Fikirkan Boleh	Metropolitan	154
Standing In The Eyes Of The World	Ella	84
Stronger	Kanye West	104
Na Na Na	My Chemical Romance	166
Smells Like Teen Spirit	Nirvana	116
Afterlife	Avenged Sevenfold	110
All I Do Is Win	DJ Khaled ft. Ludacris, Rick Ross, Snoop Dogg, and T-Pain	150
Battle Scars	Guy Sebastian ft. Lupe Fiasco	84
Worth It	Fifth Harmony ft. Kid Ink	100
Unstoppable	Sia	89
Bang Bang	Jessie J, Ariana Grande, and Nicki Minaj	150

#### Ambient Temperature, Relative Humidity and Music Volume

**FIGURE 1:**
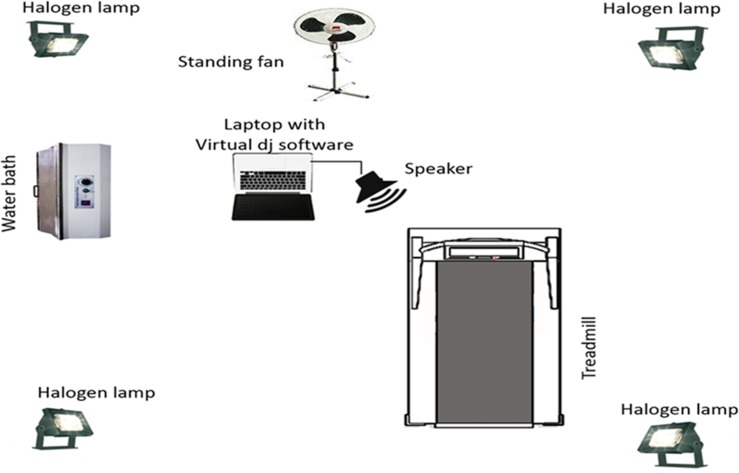
Schematic of the experimental set-up.

**Figure 1** shows a schematic of the experimental set up. An hour prior to exercise testing, the exercise laboratory was heated to 31°C using halogen lamps (Philips-500 W, France). Humidity was established at 70% using a water-bath (Memment W350t, Germany). A standing fan was used to mimic airflow in an open environment. Ambient room temperature and relative humidity were monitored continuously using a digital psychrometer kit (Extech Instrument RH305, United States). Music was played via a laptop computer using Virtual DJ software with one speaker (Sony GTK-XB90) placed 1 m in front of participants at a 45° angle, although in retrospect use of stereo headphones to deliver the music may have provided a more ecologically valid set-up. Music volume was set at 75 dB, assessed adjacent participants’ ears, as recommended by Alessio and Hutchinson (1991). No filtering of the sound occurred to, for example, control how much bass was present in the sound.

### Procedure

The study received approval from the Universiti Sains Malaysia (USM) Human Research Ethics Committee (USM/JEPeM/16020085) and was conducted in accordance with the guidelines of the International Declaration of Helsinki. The test protocol involved five sessions each of 1 h duration over a 3–5 week period. During sessions 1–3, music selection, anthropometric assessment, submaximal testing, a maximum oxygen uptake ($\dot{\text{V}}$O_2_max) test and a familiarization trial were conducted. During sessions 4–5, participants completed two counterbalanced, experimental trials running in the heat with either synchronous music or no music.

The submaximal test involved running on a motorized treadmill (Track Master TMX425CP, United States) to determine the relationship between speed and oxygen consumption. Each participant completed a 1–2 min warm-up at 5.0–6.0 km h^−1^ followed by four, 4-min periods of running at 6, 7, 8, and 9 km h^−1^. Expired air was measured at 20 s intervals using a gas analyzer (Metamax 3B, Germany). Heart rate (HR) and rate of perceived exertion (RPE) were assessed during the final min of each 4-min running period, as recommended by Minetti et al. (1994).

To assess $\dot{\text{V}}$O_2_max, participants completed a 2-min warm-up at 5.0–6.0 km h^−1^ and then rested for 1 min while headgear, mouthpiece, and nose clip were fitted. An appropriate speed was selected that allowed participants to run for at least 12 min based on previous calculations of individual speed–$\dot{\text{V}}$O_2_ correlations. The incremental test began with a gradient of 3.5°, which was increased by 2.5° at the end of each 3-min period (3.5°, 6.0°, 8.5°, 11.0°). Expired air was measured at 20 s intervals. HR and RPE were recorded in the last 30 s of each gradient increment. Data collection continued until participants raised one finger to signal the final 1 min before exhaustion. Treadmill speed was reduced rapidly and progressively after the final 1 min was completed.

During the familiarization trial, participants ran at 60% $\dot{\text{V}}$O_2_max for 60 min and then continued running at 80% of their $\dot{\text{V}}$O_2_max until exhaustion. The familiarization trial was completed to ensure that the prescribed intensity was sustainable for participants without risking injury. Running cadence (step/min) of participants was established during this trial using a GPS-enabled watch (Suunto, Ambit2S, Finland). The familiarization trial was carried out in the heat (31°C, 70% relative humidity). After the familiarization, the first experimental trial was conducted 1 week later.

Two experimental trials (synchronous music and no music) were completed in the climatic chamber. During the synchronous music condition, music was played for 60 min while running in the heat chamber at 60% $\dot{\text{V}}$O_2_max and throughout the run to exhaustion at 80% $\dot{\text{V}}$O_2_max, terminating upon exhaustion. A 1-week recovery period occurred between the two trials. Participants reported to the laboratory at 8.00am, having refrained from food consumption for at least eight hours. Participants consumed a standardized breakfast of 1 piece of white bread (60 calories) and 250 ml of plain water 2 h prior to the experiment trial.

Each experimental trial was conducted on a motorized treadmill (Track Master TMX425CP, United States) and running time-to-exhaustion time was recorded using a stopwatch. Tympanic temperature was monitored every 10 min. Heart rate was recorded at pre-, 15 min, 30 min, 45 min, 60 min, exhaustion and 1 h recovery. For hydration status, urine was sampled and body weight measured (Tanita, Japan) at pre, exhaustion and 1 h recovery to obtain percentage changes of body weight (McDermott et al., 2017) and urine specific gravity (Sper Scientific 300003C). Blood samples (∼8 ml) were taken at pre-, 60 min during exercise, exhaustion and 1 h recovery using cannulation via the antecubital veins into preservative-free tubes. Whole blood was centrifuged at 1300 RCF for 10 min, and extracted plasma was stored at −20°C for later analysis. A portion of plasma was used to analyze lactate using an enzymatic calorimetric method (EnzyChrom, BioAssays, United States). A 85 μl plasma was mixed with 15 μl buffer assay enzyme containing lactate dehydrogenase and a color-producing reagent before the absorbance was read at 565 nm at 0 min and 25 min following the start of incubation. The intra- and inter-assay coefficient variation were 4.0 and 7.2%, respectively.

### Data Analyses

**FIGURE 2:**
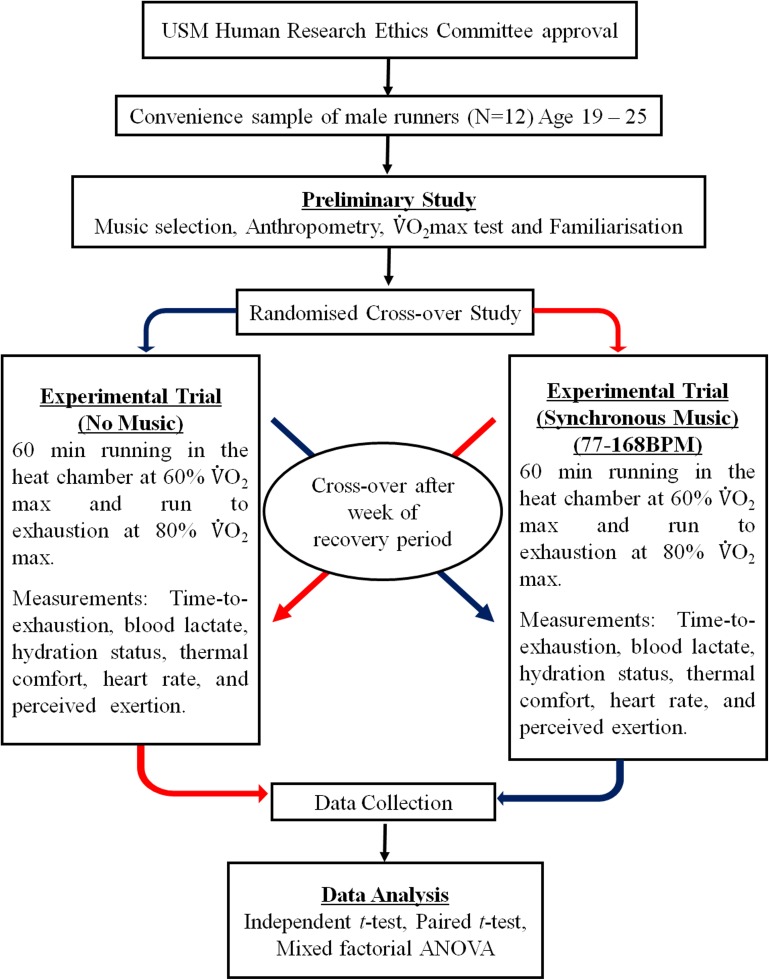
Flow chart of the study design.

Data analysis was conducted using the Statistical Package for the Social Sciences (SPSS version 22.0). Variables were described as means (*M*) ± standard deviation (*SD*), and distributions of all variables were assessed for normality. No non-normal distributions were identified. Mixed factorial ANOVAs and pairwise comparisons were conducted on HR, RPE, blood lactate, and thermal comfort data. A paired samples *t* – test was used to compare time to exhaustion between the two experimental conditions. A flow chart of the study design is shown in **Figure 2**.

## Results

### Heart Rate

**Table 2** shows the HR comparisons for 12 runners during the synchronous music and no music conditions. Mixed factorial ANOVA identified a significant difference in HR over time, *F*(6,17) = 432.64, *p* = 0.001, η^2^ = 0.993 but no significant overall difference between groups *F*(1,22) = 0.056, *p* = 0.816, η^2^ = 0.003, nor interaction between time and group, *F*(6,17) = 0.183, *p* = 0.978, η^2^ = 0.061. Pairwise comparisons showed that HR was 1–3 bpm lower for the synchronous music condition compared to the no music condition at each stage of the running trial. This difference was statistically significant at the 15 min mark (*t* = 2.20, *p* = 0.05, *d* = 0.16).

**Table 2:** Heart rate data for 12 runners under synchronous music and no music conditions.

	No music	Synchronous music	*t*	*p*-value	Effect size *(d)*
Heart Rate – pre-test (bpm)	78.08 ± 14.92	78.50 ± 9.61	−0.011	0.92	0.03
Heart Rate – 15 min (bpm)	152.67 ± 11.56	150.58 ± 13.16^∗^	2.20	0.05	0.16
Heart Rate – 30 min (bpm)	161.58 ± 12.25	158.75 ± 15.20	1.41	0.19	0.21
Heart Rate – 45 min (bpm)	167.17 ± 12.66	165.83 ± 18.42	0.56	0.59	0.08
Heart Rate – 60 min (bpm)	171.17 ± 13.69	170.67 ± 18.12	0.24	0.82	0.03
Heart Rate – exhaustion (bpm)	186.75 ± 11.50	185.50 ± 12.58	0.41	0.69	0.10
Heart Rate – 1 h recovery	85.83 ± 14.07	85.50 ± 12.95	0.11	0.92	0.02

*Data expressed as mean ± standard deviation. ^∗^p ≤ 0.05.*

### Blood Lactate

**Table 3** shows blood lactate comparisons for 12 runners during the synchronous music and no music conditions. Mixed factorial ANOVA identified a significant difference in blood lactate over time, *F*(3,20) = 30.51, *p* = 0.001, η^2^ = 0.821 but no significant difference between groups *F*(1,22) = 0.015, *p* = 0.903, η^2^ = 0.001 nor interaction between time and group, *F*(3,20) = 0.796, *p* = 0.510, η^2^ = 0.107.

**Table 3:** Blood lactate data for 12 runners under synchronous music and no music conditions.

	No music	Synchronous music	*t*	*p*-value	Effect size *(d)*
Lactate – pre-test (mmol/L)	2.98 ± 1.43	2.21 ± 1.32	1.36	0.20	0.56
Lactate – 60min (mmol/L)	3.54 ± 1.93	3.89 ± 2.64	−0.53	0.60	0.15
Lactate – exhaustion (mmol/L)	7.80 ± 2.67	8.33 ± 1.83	−0.56	0.59	0.23
Lactate recovery (mmol/L)	2.50 ± 2.71	2.58 ± 0.47	0.09	0.93	0.04

*Data expressed as mean ± standard deviation.*

### Hydration Status

**Table 4** shows urine specific gravity and % body weight change for 12 runners during the synchronous music and no music conditions. Mixed factorial ANOVA identified no significant differences in hydration status over time, *F*(1,22) = 1.195, *p* = 0.286, η^2^ = 0.052, no significant difference between groups, *F*(1,22) = 0.171, *p* = 0.683, η^2^ = 0.008, and no significant interaction between time and group, *F*(1,22) = 0.531, *p* = 0.474, η^2^ = 0.024.

**Table 4:** Urine specific gravity and % body weight change for 12 runners.

	No Music	Synchronous Music	*t*	*p*-value	Effect size *(d)*
Urine Specific Gravity					
Pre	1.019 ± 0.008	1.021 ± 0.009	0.76	0.46	0.23
Post	1.021 ± 0.007	1.022 ± 0.002	0.36	0.73	0.19
Pre-Post	−0.001 ± 0.003	−0.000 ± 0.004	0.83	0.43	0.28
Body weight change (%)	1.77 ± 0.57	1.82 ± 0.64	−0.55	0.59	0.08

*Data expressed as mean ± standard deviation. Body weight change (%) = [(pre-exercise bodyweight – post-exercise body weight)/pre-exercise body weight] × 100.*

### Rating of Perceived Exertion (RPE)

**Table 5** shows RPE values for 12 runners during the synchronous music and no music conditions. Mixed factorial ANOVA identified a significant difference in perceived exertion over time, *F*(5,18) = 63.234, *p* = 0.001, η^2^ = 0.946, and between groups, *F*(1,22) = 4.690, *p* = 0.041, η^2^ = 0.176, but no significant interaction between time and group, *F*(5,18) = 1.117, *p* = 0.386, η^2^ = 0.237. RPE was significantly lower in the synchronous music condition throughout the steady state portions of the running trial, (*d* = 0.72 – 1.05) even though the workload was objectively the same. During the first 60 min of the two trials, when participants completed the same workload, RPE was on average 22% lower for the synchronous music condition. Perceived exertion at the end of the run-to-exhaustion was 5% lower (*d* = 0.34) for the synchronous music condition even though participants had run for significantly longer at the same intensity.

**Table 5:** RPE for 12 runners under synchronous music and no music conditions.

	No Music	Synchronous Music	*t*	*p*-value	Effect size *(d)*
RPE – pre-test	6.83 ± 1.59	6.50 ± 0.90	1.30	0.22	0.26
RPE – 15 min	9.92 ± 2.57	7.67 ± 1.61^∗∗^	3.04	0.01	1.05
RPE – 30 min	11.17 ± 2.48	8.83 ± 2.55^∗∗^	3.39	0.01	0.93
RPE – 45 min	13.08 ± 2.15	11.00 ± 3.05^∗∗^	3.49	0.01	0.79
RPE – 60 min	14.58 ± 2.23	12.83 ± 2.59^∗∗^	3.17	0.01	0.72
RPE - exhaustion	17.83 ± 2.08	17.00 ± 2.80	1.36	0.20	0.34

*Data expressed as mean ± standard deviation. ^∗∗^p ≤ 0.01.*

### Thermal Comfort

**Table 6** shows thermal comfort scores for 12 runners during the synchronous music and no music conditions. Mixed factorial ANOVA identified no significant difference in thermal comfort over time, *F*(4,19) = 18.680, *p* = 0.001, η^2^ = 0.797, no significant difference between groups, *F*(1,22) = 0.007, *p* = 0.934, η^2^ = 0.918, and no significant interaction between time and group, *F*(4,19) = 1.579, *p* = 0.221, η^2^ = 0.249.

**Table 6:** Thermal comfort scores for 12 runners under synchronous music and no music conditions.

	No Music	Synchronous Music	*t*	*p*-value	Effect size *(d)*
Thermal Comfort – pre-test	0.42 ± 0.67	0.25 ± 0.97	1.00	0.34	0.20
Thermal Comfort – 15 min	1.33 ± 0.65	1.33 ± 0.49	0.01	1.00	0.0
Thermal Comfort – 30 min	1.58 ± 0.79	1.75 ± 0.75	−0.80	0.44	0.22
Thermal Comfort – 45 min	2.17 ± 0.72	1.92 ± 0.79	1.39	0.19	0.33
Thermal Comfort – 60 min	2.33 ± 0.98	2.5 ± 0.80	−0.69	0.50	0.19

*Data expressed as mean ± standard deviation.*

### Running Time-to-Exhaustion

**Table 7** shows running time-to-exhaustion for 12 runners during the synchronous music and no music conditions. Participants ran for significantly longer before reaching exhaustion while listening to synchronous music (376.50 ± 304.97 s) compared to the no music condition (226 ± 150.32 s), *t* = 2.63, *p* = 0.02. The performance benefit of running to synchronous music represented a moderate-to-large effect (*d* = 0.63), with a mean gain of 150.50 s.

**Table 7:** Time-to-exhaustion for 12 runners under synchronous music and no music conditions.

	No music	Synchronous music	*t*	*p*-value	Effect size *(d)*
Time-to-exhaustion (s)	226 ± 150.32	376.50 ± 304.97^∗^	−2.63	0.02	0.63

*Data expressed as mean ± standard deviation. ^∗^p ≤ 0.05.*

## Discussion

The present study investigated effects of listening to synchronous music on physiological, psychophysiological and performance measures under heat stress conditions. The greatest benefit of synchronous music was apparent for time-to-exhaustion, where participants ran 2.5 min longer on average, equating to a 66.59% improvement over the no music condition. This performance benefit is consistent with previous findings (e.g., Karageorghis et al., 2009; Terry et al., 2012) but of a much greater magnitude. In addition, perceived exertion was significantly lower, by an average of 22%, during the synchronous music condition compared to the no music condition. This benefit is also consistent with previous findings (e.g., Dyrlund and Wininger, 2008; Hutchinson and Karageorghis, 2013) but again is of a greater magnitude.

The mechanisms by which listening to music could improve endurance performance by such a substantial margin are not clearly established, although several factors may have contributed in combination. Firstly, a benefit may accrue via attentional processes, wherein limited processing capacity causes signals of fatigue to be masked by attending to music, thereby reducing perceived exertion and encouraging participants to work harder and/or for longer (Hardy and Rejeski, 1989). This effect would tend to be nullified toward the latter stages of a run-to-exhaustion because the intensity of the fatigue symptoms, particularly the sharp rise in respiration rate and blood lactate, diverts attention away from the music (Ekkekakis, 2003).

Secondly, from an evolutionary perspective, humans appear to have gained a genetic predisposition to synchronize movement to musical rhythms (Patel, 2008; Phillips-Silver and Keller, 2012). A central pattern generator or pacemaker in the brain has been proposed (Schneider et al., 2010), which regulates temporal functioning and governs the human rhythm response, meaning that enhanced rhythm in the movement pattern created by running synchronously to music helps to coordinate nerve signal processes that regulate locomotion, neurovascular control and sensory integration more efficiently. Finally, exercisers have reported that running in synchrony with the prominent beat of a music track can create feelings that border on a spiritual experience, which appear to inspire them to additional effort (Juslin, 2013).

Perceived exertion was significantly lower during the synchronous music condition at each stage of the running trials, which is consistent with previous research where participants completed the same workload with and without music (e.g., Dyrlund and Wininger, 2008; Hutchinson and Karageorghis, 2013). In the absence of the external stimulation of the music, participants may have focused more on their own efforts, detecting increased signals of fatigue in the process (Edworthy and Waring, 2006). Unsolicited feedback from participants indicated that thoughts of the time remaining and the required exertion prevailed during the no music trial, whereas during the synchronous music condition the beat of the music distracted them from the effort of running, and assisted continuation due to the motivational qualities of the songs.

Heart rate was 1–3 bpm lower for synchronous music at each time point during the running trial and at exhaustion. This difference was significant at the 15-min mark. It is sometimes unclear whether decreased HR represents an advantage or disadvantage of music. With consistent workload across conditions, such as during the first 60 min of the running trials in our study, lower HR represents a benefit of music, albeit small in this case. However, where participants attempt to produce maximal power (Stork et al., 2015), go faster (Tate et al., 2012), or maintain effort for longer (Bood et al., 2013), interpretation is more challenging. A benefit ensues if HR is lower despite equivalent or greater workload, whereas a disadvantage ensues if HR is higher despite equivalent or lesser workload. However, where HR is higher with a greater workload (Eliakim et al., 2007; Sanchez et al., 2014), it is unclear whether this is indicative of an advantage, given that increased HR may be a function of additional work rather than the effect of music. Researchers should consider this potential confounding effect when designing studies to test the influence of music on HR and other physiological parameters.

In our study, the lower HR values for the synchronous music condition compared to no music during the first 60 min of the run, despite running speed being objectively the same for both conditions, suggests that music had some capacity to improve running economy. This may have occurred because running in synchrony with music required fewer micro adjustments to running stride resulting in a more biomechanically efficient run. Alternatively, music may have promoted a generalized relaxation response in participants that marginally enhanced blood flow efficiency. Another possibility is that music may have facilitated the entrainment process (Clayton, 2012) whereby the runners entrained their breathing pattern to their stride rate.

No significant between-group differences were found for blood lactate, hydration status, or thermal comfort. Previous studies assessing blood lactate accumulation during steady state endurance performance with and without music (Schwartz et al., 1990; Szmedra and Bacharach, 1998; Terry et al., 2012) provided equivocal findings. Our results suggest that synchronous music offers no benefit to lactate production during endurance activity in hot and humid conditions. Hydration status, assessed in terms of urine specific gravity and percentage body weight change, also did not vary between conditions, indicating that synchronizing running stride to music tempo offered no benefit in terms of ameliorating hypohydration. Thermal comfort was assessed in the present study primarily for safety reasons. Given that the run-to-exhaustion was completed under heat stress, monitoring thermal comfort was a condition of ethics approval. Unsurprisingly, results showed no difference in thermal comfort ratings because temperature and humidity were identical for both the synchronous music and no music conditions. This finding indicated that synchronous music did not distract participants from sensations of heat and humidity.

## Conclusion

Results showed that running in hot and humid conditions while listening to synchronous music significantly improved running time-to-exhaustion, lowered perceived exertion and, to a lesser extent, reduced HR. Findings have application for runners performing in tropical conditions.

## Author Contributions

All authors listed have made a substantial, direct and intellectual contribution to the work, and approved it for publication.

## Conflict of Interest Statement

The authors declare that the research was conducted in the absence of any commercial or financial relationships that could be construed as a potential conflict of interest.
